# Correction: 16th International Congress on Early Onset Scoliosis

**DOI:** 10.1007/s43390-022-00616-3

**Published:** 2022-12-16

## Correction: Spine Deforms 10.1007/s43390-022-00597-3

Pediatric Spine Foundation

The article 16th International Congress on Early Onset Scoliosis by the Pediatric Spine Foundation, was originally published electronically on the publisher’s internet portal on Published 06 October 2022 without open access. With the foundations decision to opt for Open Choice the copyright of the article changed on 11.11.22 to © The Authors 2022 and the article is forthwith distributed under a Creative Commons Attribution 4.0 International License, which permits use, sharing, adaptation, distribution and reproduction in any medium or format, as long as you give appropriate credit to the original author(s) and the source, provide a link to the Creative Commons licence, and indicate if changes were made.

The images or other third party material in this article are included in the article’s Creative Commons licence, unless indicated otherwise in a credit line to the material. If material is not included in the article’s Creative Commons licence and your intended use is not permitted by statutory regulation or exceeds the permitted use, you will need to obtain permission directly from the copyright holder.

To view a copy of this licence, visit http://creativecommons.org/licenses/by/4.0/.

Furthermore, the following corrections and additions have been made to the article: 16th International Congress on Early Onset Scoliosis. Spine Deform (2022). https://doi.org/10.1007/s43390-022-00597-3. Published 06 October 2022.

Publisher apologizes for the inconvenience caused.

## Paper 8

### Is spine straightness correlated with mental health in early-onset scoliosis patients treated with growing rods? Psychological assessment in graduated patients

#### Yildiz Mevhibe Irem, Barlas Goker, Talat Demirsoz, Cihan Aslan, Halil Gokhan Demirkiran, Sevilay Karahan, Mumin Kazim Yazici, Muharrem Yazic

The following table, to which the text refers, has been added:
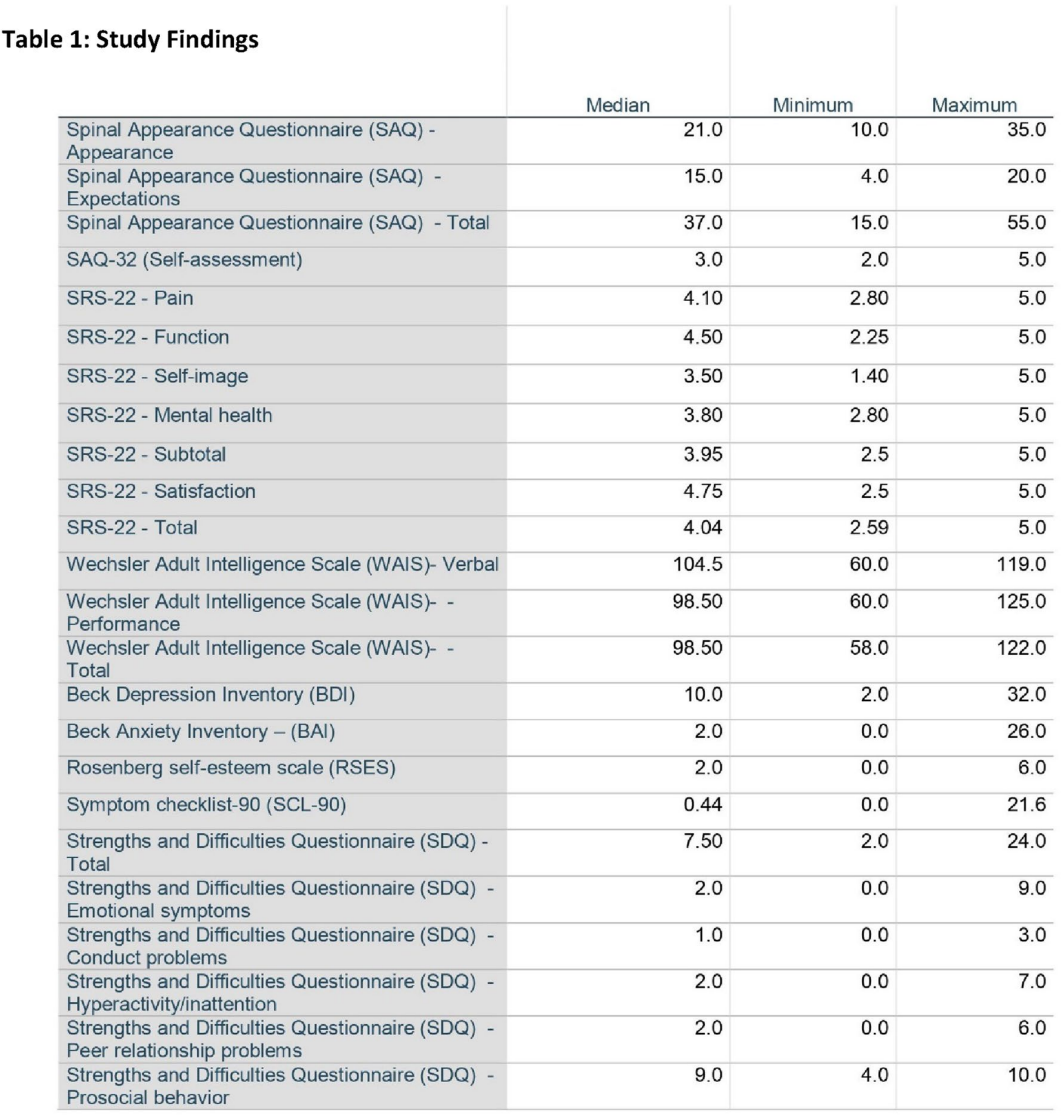


## Paper 9

### There is no magic to it: lengthening behavior of magnetically controlled growing rods in early onset scoliosis

#### Jessica H. Heyer, Jason B. Anari, Stuart L. Mitchell, Keith D. Baldwin, John M. Flynn, MD, Pediatric Spine Study Group, n/a, Patrick J. Cahill, MD

The following figures have been added:
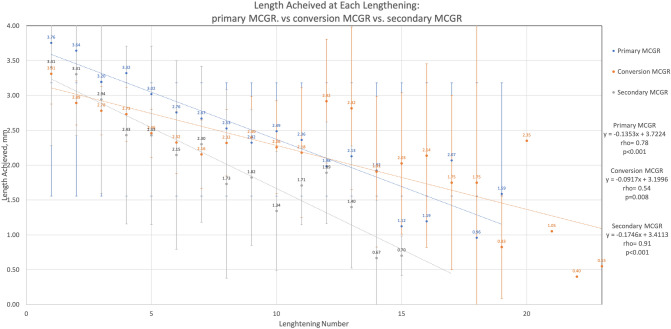



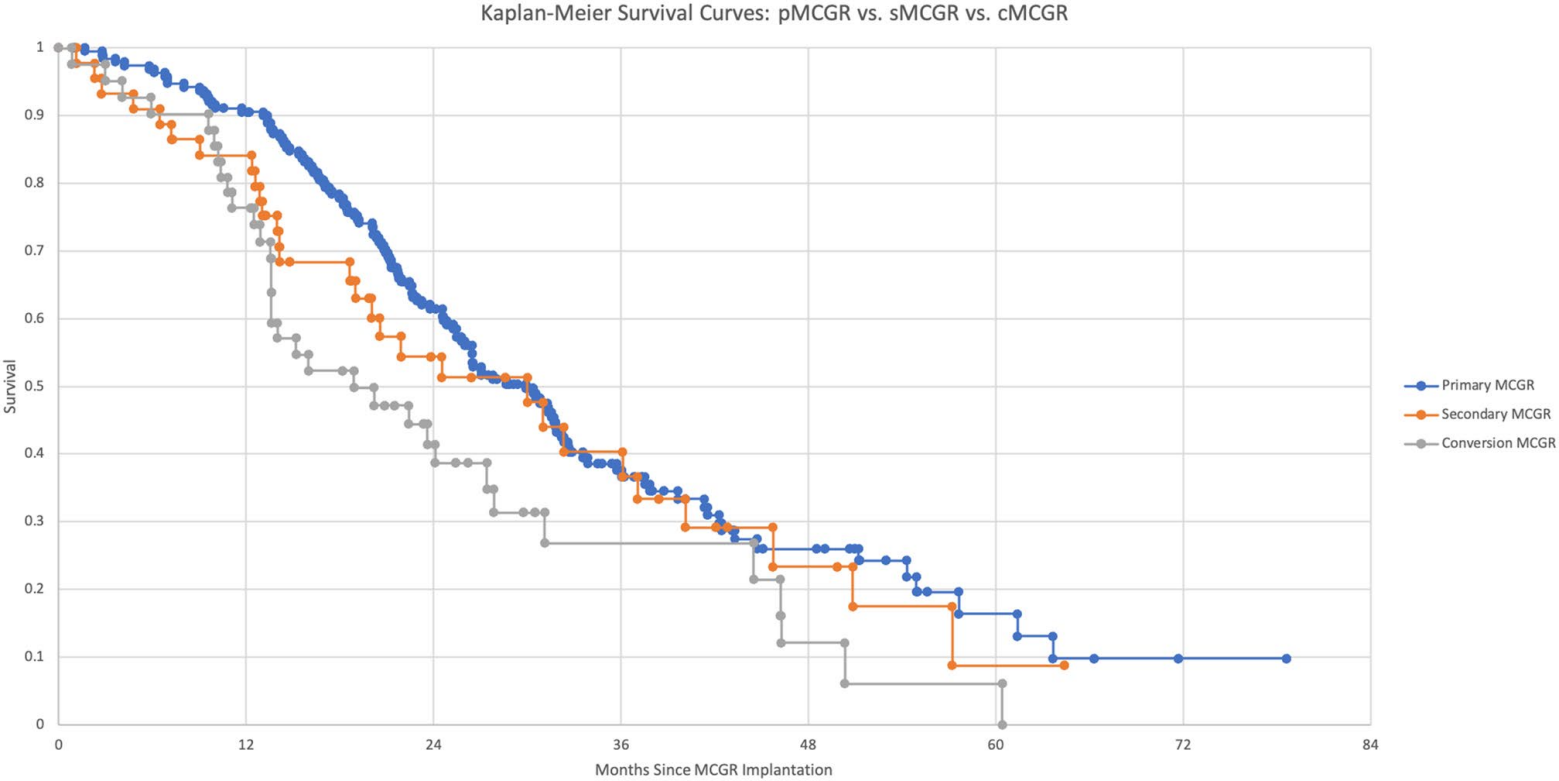


The following uploaded file has been removed:

- Length Achieved at Each Lengthening, Primary MCGR vs. Conversion MCGR vs. Secondary MCGR

Papers 14 and 31 have been replaced by two new papers.

## Paper 14

### Outcomes of traditional dual growing rods with apical control techniques for the treatment of early onset scoliosis—a comparison with TDGR only during lengthening procedures ‡ 3 years after index surgery

#### Shengru Wang, Terry Jianguo Zhang, Yang Yang, You Du, Yiwei Zhao

is replaced by the paper:

### Growth Guidance Surgery with Fusion at Upper Foundation and Segmental Guidance Screwing at Lower Foundation (modified Shilla Surgery) for Early-onset Scoliosis: Evaluation at Least 2 Years Follow Up

#### Masaaki Ito, Koki Uno, Teppei Suzuki

Introduction: Although the number of total surgeries of growth guidance surgery with apex fusion (Shilla surgery) was far less small comparing TGR, complication rate was comparable to that of TGR due to dislodgements at upper and lower foundation and controlling kyphosis has been a concern. To avoid these problems, we fused upper foundation with apex and performed segmental guiding screwing at lower foundation. It is a method in which the proximal thoracic spine is fused and growth in the caudal direction is allowed. The purpose of this study is to examine the preliminary 2 years outcome of this new strategy after initial surgery.

Objective: Growth guidance surgery with fusion at upper foundation and segmental guiding screwing at lower foundation (modified Shilla) might reduce unplanned surgery and have acceptable radiological outcome during growth guidance period.

Methods: We examined consecutive 51 cases who underwent Shilla growth guidance for EOS more than 2 years after surgery. There were 38 cases (male 12) of conventional Shilla growth guidance surgery (group C) and 13 cases (male 5) of modified Shilla growth guidance surgery (group M). In group M, the upper thoracic vertebra (T2-4) is exposed and the pedicle screw is inserted for fusion. For the apex of spine, transverse anchors were used without exposing the facet joints, and about 8–10 pedicle screws were inserted from the lower thoracic to the lumbar vertebra under the fluoroscopy using Wiltse paraspinal approach. The radiological outcomes (Cobb angles of scoliosis and kyphosis, T1-T12 height, T1-S1 length), and the number of complications from the initial surgery to the final follow-up were examined.

Results: The average age at the initial surgery and the follow-up period were 7.9 y.o and 5.2 years in group C, 8.4 y.o and 2.4 years in group M, respectively. The follow-up period was significantly longer in group C (The unplanned surgeries until 2 years after surgery were required for 7 cases in group C, on the other hand, there is no unplanned surgery in group M. The distal implant loosening or dislodgement was seen 23 case (61%) in group C and 2 cases (15.4%) in group M, which were significantly lower in group M.

Conclusion: The modified Shilla growth guidance surgery showed less correction loss of the main thoracic curve and a larger T1-S1 length with less complications comparing to conventional Shilla growth guidance surgery at preliminary 2 years follow-up after the initial surgery. Modified Shilla growth guidance surgery using segmental guiding pedicle screws on the distal side of the spine may reduce unplanned surgery due to the distal implant problems.

## Paper 31

### The effect of pedicle screw instrumentation at a young age on upper thoracic vertebra and canal development

#### Nan Wu, Terry Jianguo Zhang, Shengru Wang, Lian Liu

is replaced by the paper:

### Utility of a Risk Severity Scoring System to Predict Infections in Patients Undergoing Growth Guidance Surgery for Early Onset Scoliosis

#### Adam Jamnik, Anna McClung-Booth, David Thornberg, Pediatric Spine Study Group, Brandon Ramo

Introduction: Of patients with Prader-Willi Syndrome (PWS), as many as 70% develop scoliosis before skeletal maturity, and approximately 15% of all PWS patients eventually require surgery for their spinal deformity. These patients have been noted to experience particularly high surgical complication rates, likely in part due to skin-picking causing an increased risk for infection as well as muscular and bony abnormalities increasing the need for implant-associated re-operation. A single prior paper on 13 PWS patients undergoing growth friendly surgery demonstrated a complication rate of 2.2 complications per patient at the 2 year follow-up point. Given the high complication rate of growth-friendly surgery in any patient, further study into the risks of growth-friendly surgery in PWS patients is needed.

Aims/Objectives: This study aimed to further elucidate the intermediate-term complication profile associated with growth-friendly surgery in PWS patients by assessing surgical correction of the deformity and complications associated with treatment at minimum 4-year follow-up.

Methods A retrospective review of patients in the multicenter database maintained by the Pediatric Spine Study Group was performed. All patients with PWS that underwent surgery with Traditional Growing Rods (TGR), Magnetically Controlled Growing Rods (MCGR), Vertical Expandable Prosthetic Titanium Rib (VEPTR), or Luque Trolley with > 4 year follow up were included in this study. Patients were excluded if they did not have sufficient follow-up information in the multicenter database. Unplanned Returns to the Operating Room (UPROR), as well as non-operative complications, were recorded for all patients starting from the date of their initial growth-friendly scoliosis surgery.

Results: Twenty-two patients met the initial inclusion criteria. Eight patients were excluded because they were missing the radiographs necessary for analysis, leaving 14 patients with adequate follow-up and radiographs to be analyzed. The average age at index surgery was 5.34 ± 1.84 years, and 8 (57.1%) of the patients were female. In total, 10/14 patients (71%) experienced at least one UPROR, with a total of 23 UPRORs in those 10 patients. Twenty-one of these complications were related to implant failure, one was infectious, and one was neurologic. The timing and frequency of these UPRORs are shown in Table 1. The distribution of complications by type of surgical implant during index scoliosis surgery is shown in Table 2.

Conclusions: Growth-friendly surgery in patients with PWS is associated with high rates of implant failure requiring unplanned return to the operating room. Our determined rate of 21 implant failures requiring UPROR in 14 patients (average of 1.5 per patient) was significantly higher than the average of 0.6 implant-related UPROR per patient that underwent definitive fusion in a separate study of the same disease etiology (mean age at surgery 12.3; Accadbled et al., 2008). This study also found a lower rate of infections than previous studies of PWS patients, though this may be due to limitations in data availability for patients in the multicenter database. Although the small sample size prevents true statistical comparison, the frequency of patients experiencing UPROR appears to be independent of the type of instrumentation used.
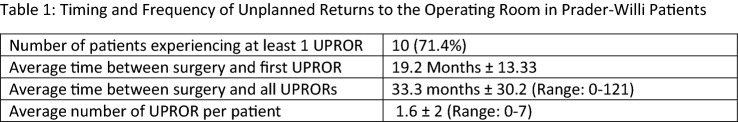



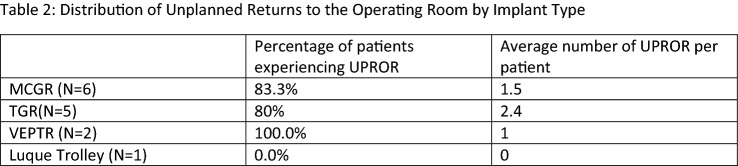


**Author Disclosures**

The following Disclosures have been added:

Adam Jamnik—Nothing to Disclose.

Pediatric Spine Study Group—Does Disclose Boston Orthotics & Prosthetics: Educational support|DePuy Synthes Spine: Grant/Research Support|Globus Medical: Grant/Research Support|Medtronic: Grant/Research Support|NuVasive: Grant/Research Support|nView.

